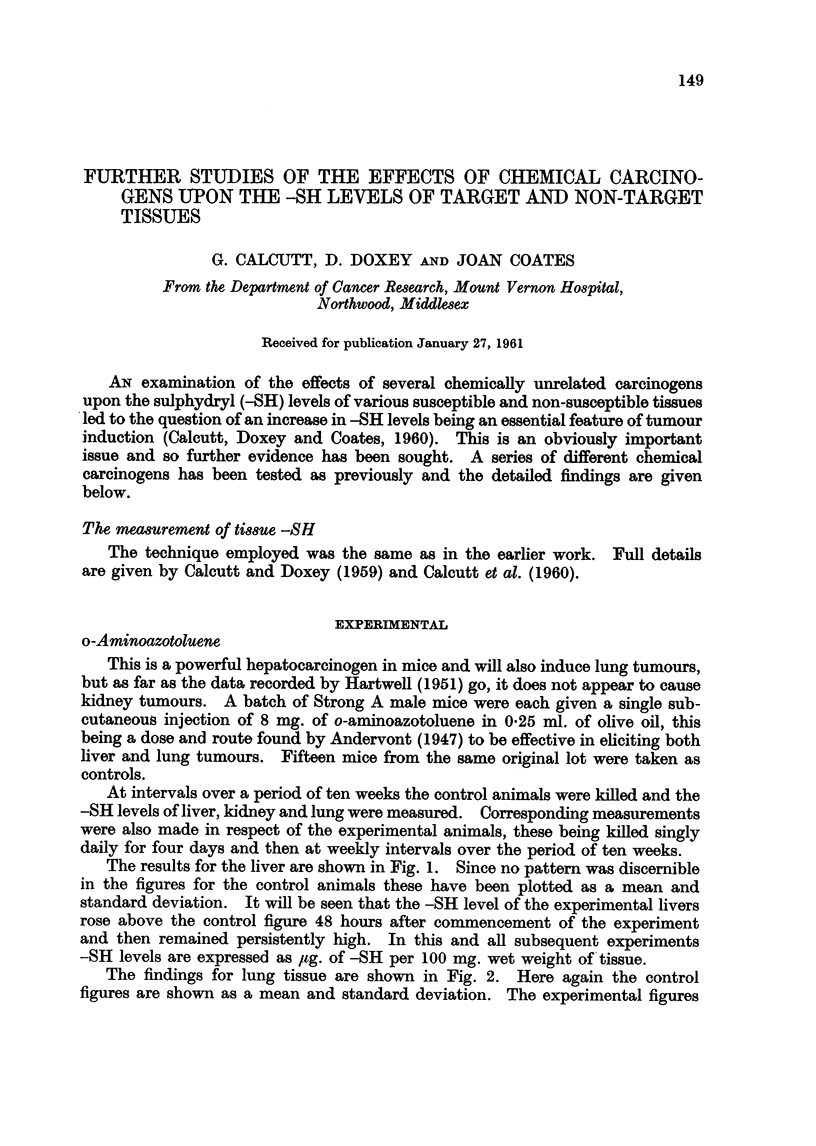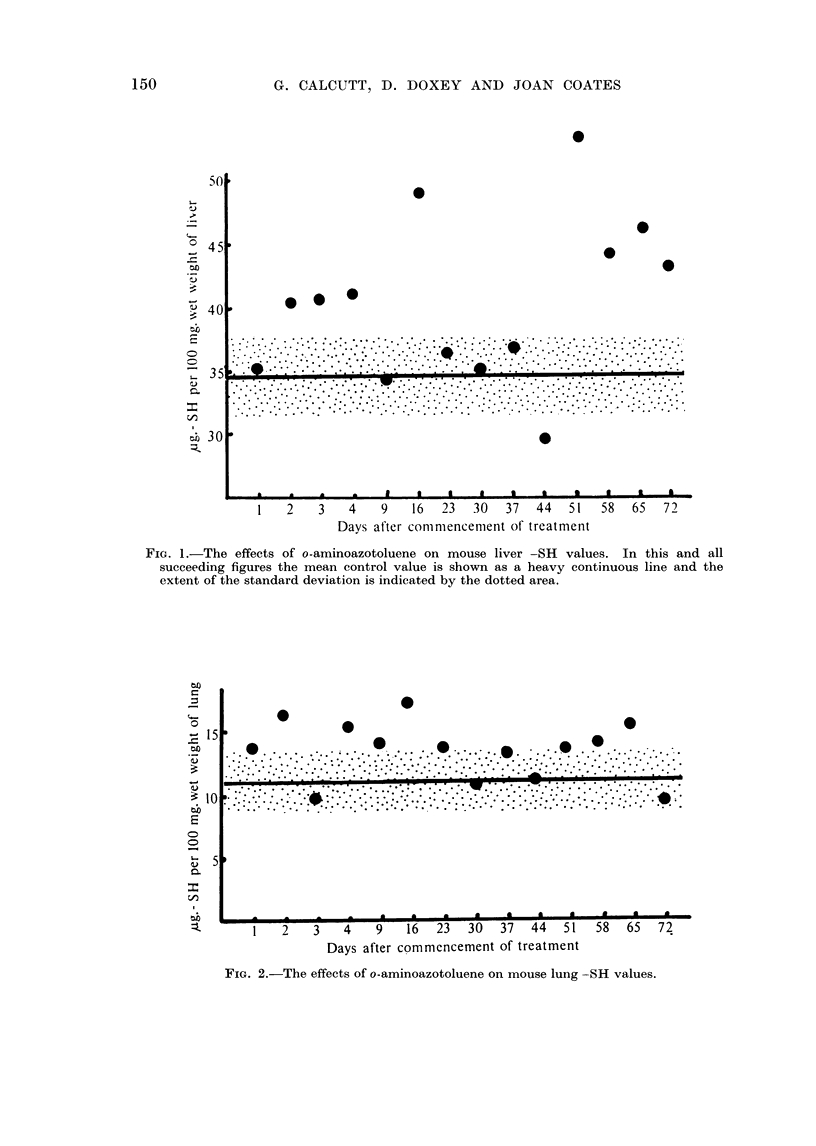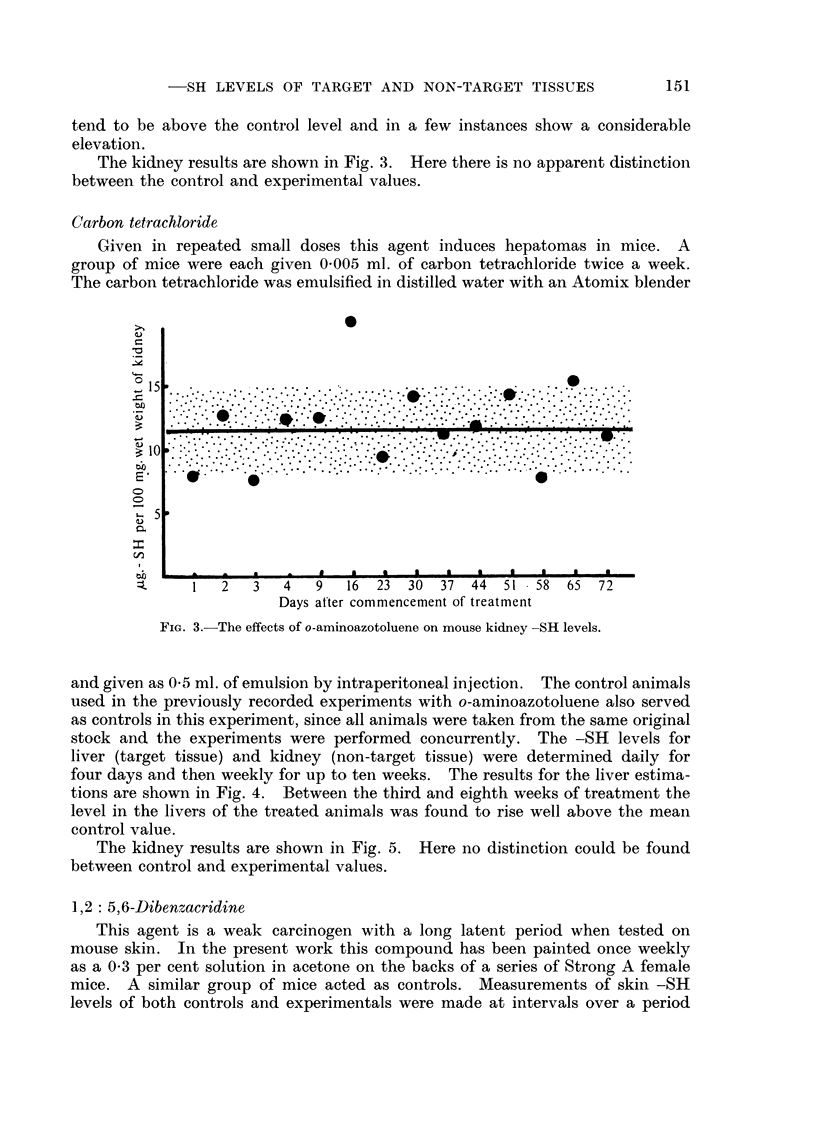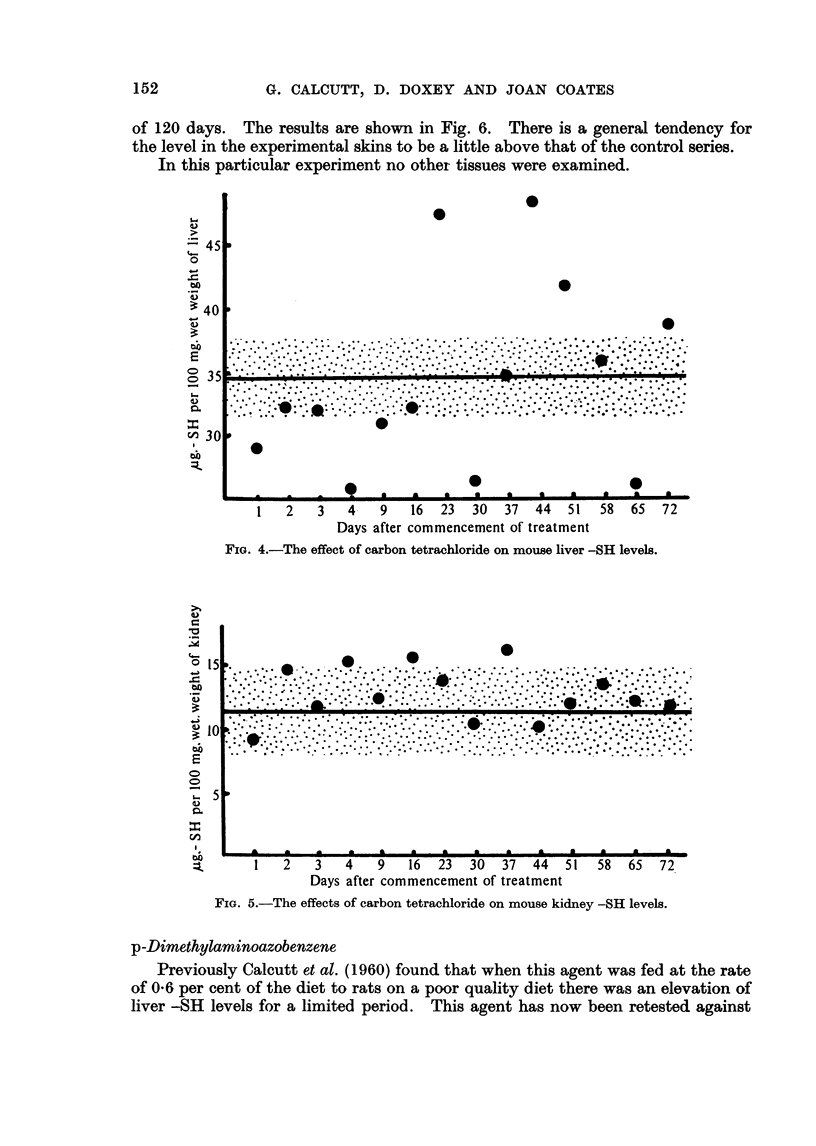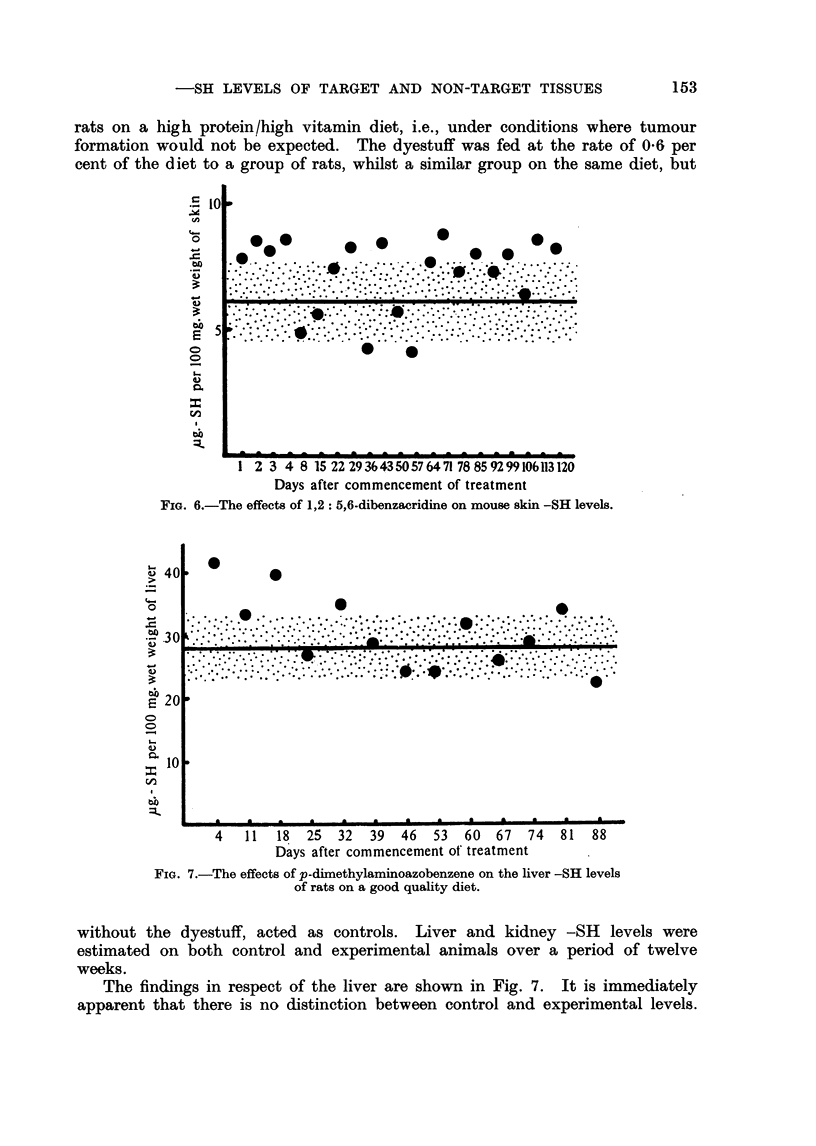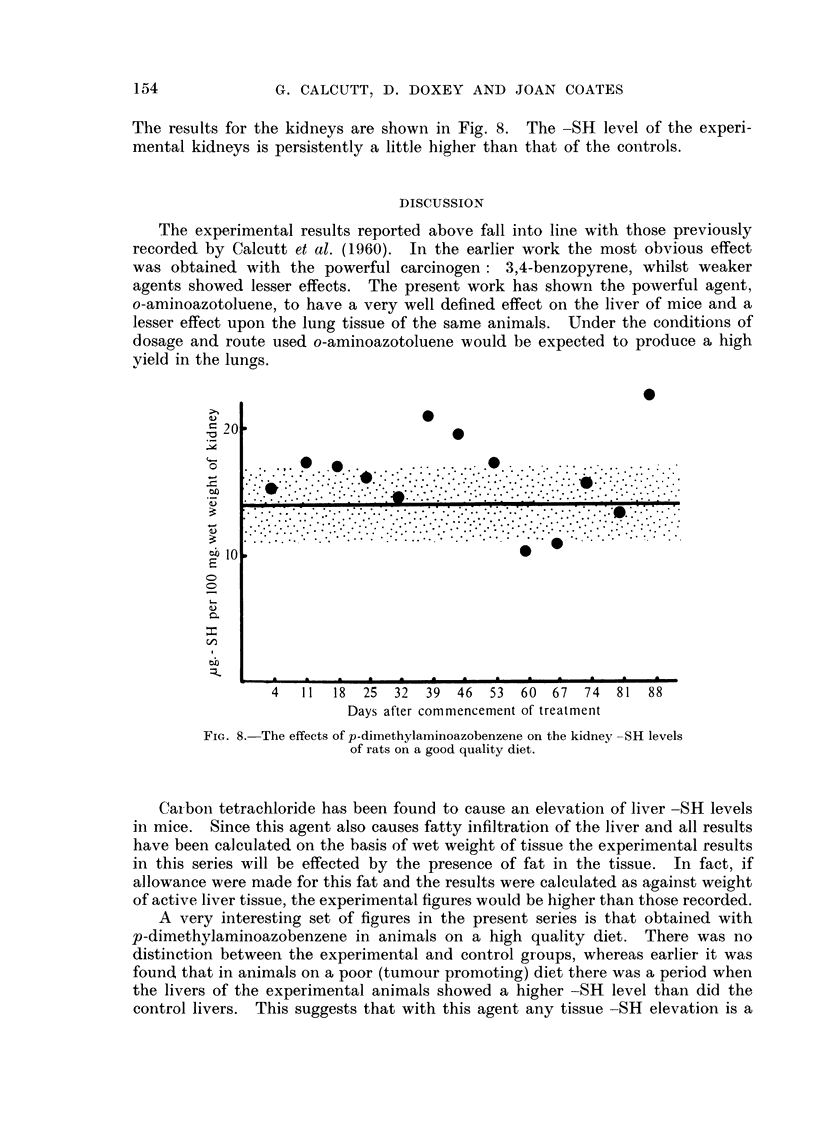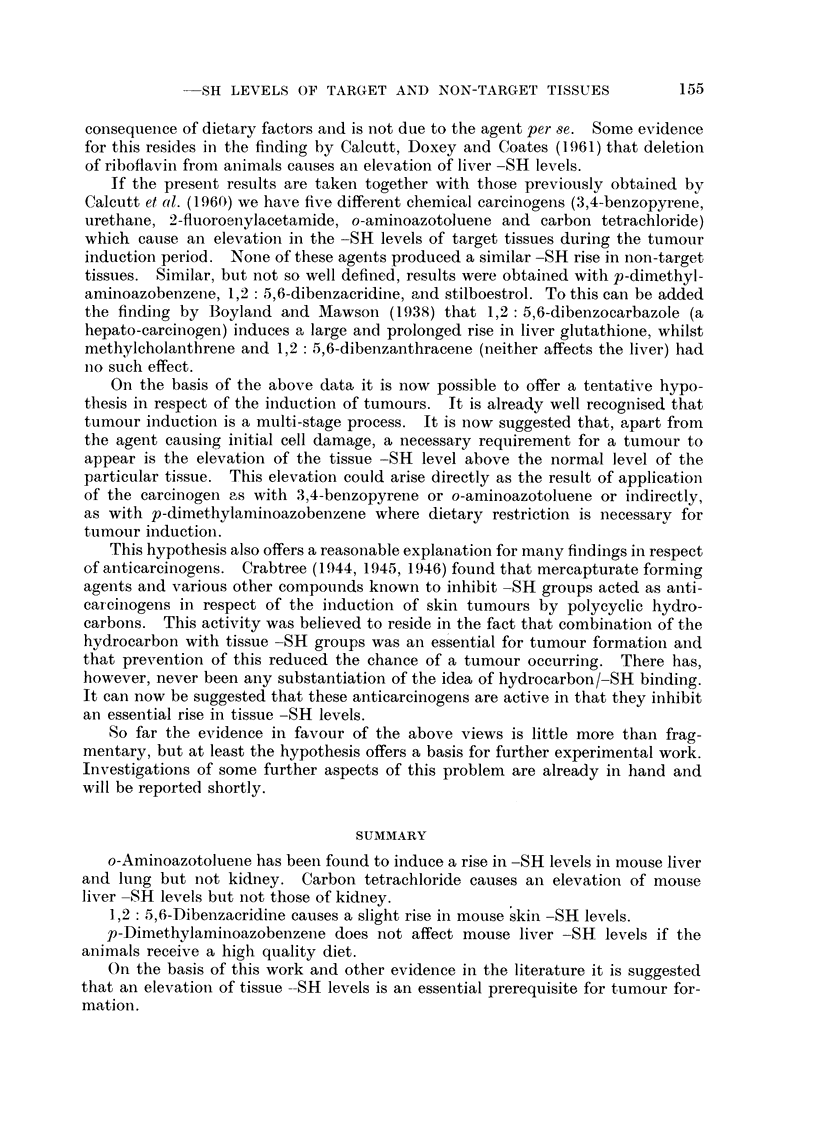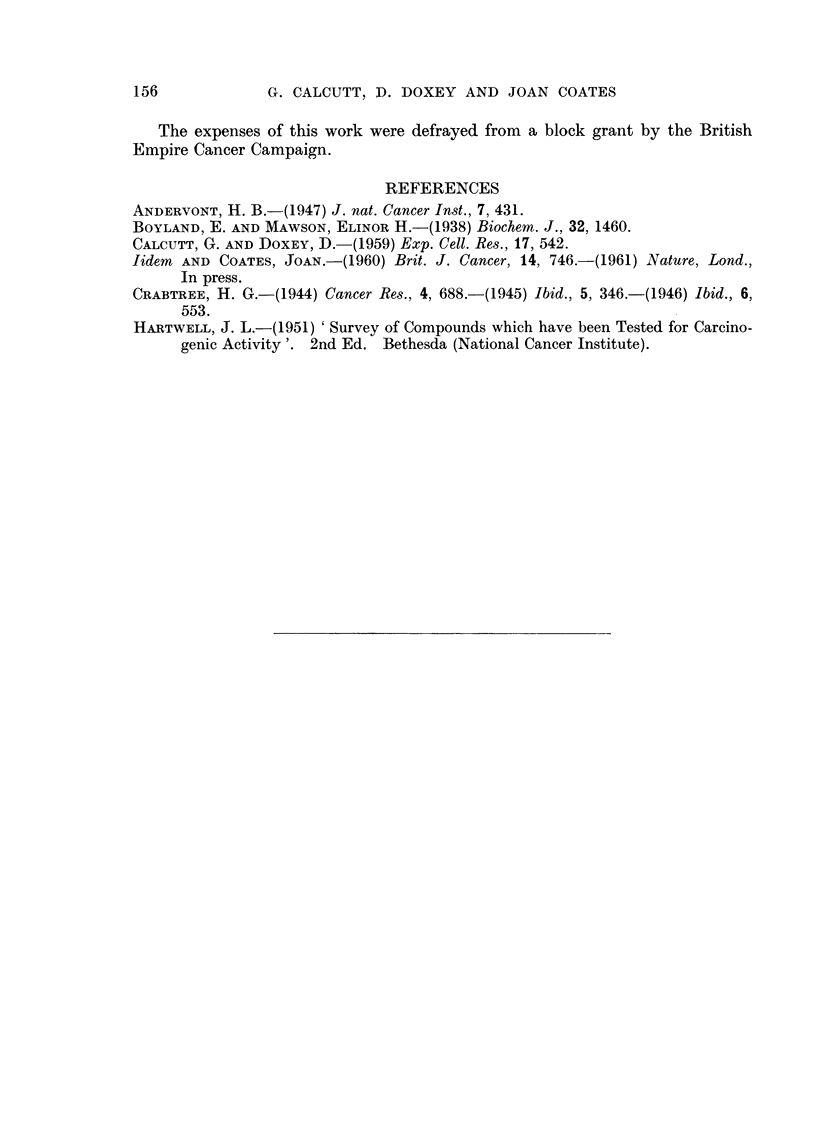# Further Studies of the Effects of Chemical Carcinogens Upon The -Sh Levels of Target and Non-Target Tissues

**DOI:** 10.1038/bjc.1961.19

**Published:** 1961-03

**Authors:** G. Calcutt, D. Doxey, Joan Coates


### FURTHER STUDIES OF THE EFFECTS OF CHEMICAL CARCINOGENS UPON THE –SH LEVELS OF TARGET AND NON-TARGET TISSUES

G. CALCUTT, D. DOXEY AND JOAN COATES

*From the Department of Cancer Research, Mount Vernon Hospital, Northwood, Middlesex*

Received for publication January 27, 1961

AN examination of the effects of several chemically unrelated carcinogens upon the sulphydryl (–SH) levels of various susceptible and non-susceptible tissues led to the question of an increase in –SH levels being an essential feature of tumour induction (Calcutt, Doxey and Coates, 1960). This is an obviously important issue and so further evidence has been sought. A series of different chemical carcinogens has been tested as previously and the detailed findings are given below.

*The measurement of tissue –SH*

The technique employed was the same as in the earlier work. Full details are given by Calcutt and Doxey (1959) and Calcutt *et al.* (1960).

### EXPERIMENTAL

o-*Aminoazotoluene*

This is a powerful hepatocarcinogen in mice and will also induce lung tumours, but as far as the data recorded by Hartwell (1951) go, it does not appear to cause kidney tumours. A batch of Strong A male mice were each given a single subcutaneous injection of 8 mg. of *o*-aminoazotoluene in 0·25 ml. of olive oil, this being a dose and route found by Andervont (1947) to be effective in eliciting both liver and lung tumours. Fifteen mice from the same original lot were taken as controls.

At intervals over a period of ten weeks the control animals were killed and the –SH levels of liver, kidney and lung were measured. Corresponding measurements were also made in respect of the experimental animals, these being killed singly daily for four days and then at weekly intervals over the period of ten weeks.

The results for the liver are shown in Fig. 1. Since no pattern was discernible in the figures for the control animals these have been plotted as a mean and standard deviation. It will be seen that the –SH level of the experimental livers rose above the control figure 48 hours after commencement of the experiment and then remained persistently high. In this and all subsequent experiments –SH levels are expressed as $\mu$g. of –SH per 100 mg. wet weight of tissue.

The findings for lung tissue are shown in Fig. 2. Here again the control figures are shown as a mean and standard deviation. The experimental figures

FIG. 1.—The effects of *o*-aminoazotoluene on mouse liver -SH values. In this and all succeeding figures the mean control value is shown as a heavy continuous line and the extent of the standard deviation is indicated by the dotted area.

FIG. 2.—The effects of *o*-aminoazotoluene on mouse lung -SH values.

tend to be above the control level and in a few instances show a considerable elevation.

The kidney results are shown in Fig. 3. Here there is no apparent distinction between the control and experimental values.

*Carbon tetrachloride*

Given in repeated small doses this agent induces hepatomas in mice. A group of mice were each given 0·005 ml. of carbon tetrachloride twice a week. The carbon tetrachloride was emulsified in distilled water with an Atomix blender

FIG. 3.—The effects of *o*-aminoazotoluene on mouse kidney –SH levels.

and given as 0·5 ml. of emulsion by intraperitoneal injection. The control animals used in the previously recorded experiments with *o*-aminoazotoluene also served as controls in this experiment, since all animals were taken from the same original stock and the experiments were performed concurrently. The –SH levels for liver (target tissue) and kidney (non-target tissue) were determined daily for four days and then weekly for up to ten weeks. The results for the liver estimations are shown in Fig. 4. Between the third and eighth weeks of treatment the level in the livers of the treated animals was found to rise well above the mean control value.

The kidney results are shown in Fig. 5. Here no distinction could be found between control and experimental values.

1,2 : 5,6-*Dibenzacridine*

This agent is a weak carcinogen with a long latent period when tested on mouse skin. In the present work this compound has been painted once weekly as a 0·3 per cent solution in acetone on the backs of a series of Strong A female mice. A similar group of mice acted as controls. Measurements of skin –SH levels of both controls and experimentals were made at intervals over a period of 120 days. The results are shown in Fig. 6. There is a general tendency for the level in the experimental skins to be a little above that of the control series.

In this particular experiment no other tissues were examined.

FIG. 4.—The effect of carbon tetrachloride on mouse liver –SH levels.

FIG. 5.—The effects of carbon tetrachloride on mouse kidney –SH levels.

p-*Dimethylaminoazobenzene*

Previously Calcutt *et al.* (1960) found that when this agent was fed at the rate of 0·6 per cent of the diet to rats on a poor quality diet there was an elevation of liver –SH levels for a limited period. This agent has now been retested against rats on a high protein/high vitamin diet, i.e., under conditions where tumour formation would not be expected. The dyestuff was fed at the rate of 0·6 per cent of the diet to a group of rats, whilst a similar group on the same diet, but

FIG. 6.—The effects of 1,2 : 5,6-dibenzacridine on mouse skin –SH levels.

FIG. 7.—The effects of *p*-dimethylaminoazobenzene on the liver –SH levels of rats on a good quality diet.

without the dyestuff, acted as controls. Liver and kidney –SH levels were estimated on both control and experimental animals over a period of twelve weeks.

The findings in respect of the liver are shown in Fig. 7. It is immediately apparent that there is no distinction between control and experimental levels.

The results for the kidneys are shown in Fig. 8. The –SH level of the experimental kidneys is persistently a little higher than that of the controls.

### DISCUSSION

The experimental results reported above fall into line with those previously recorded by Calcutt *et al.* (1960). In the earlier work the most obvious effect was obtained with the powerful carcinogen : 3,4-benzopyrene, whilst weaker agents showed lesser effects. The present work has shown the powerful agent, *o*-aminoazotoluene, to have a very well defined effect on the liver of mice and a lesser effect upon the lung tissue of the same animals. Under the conditions of dosage and route used *o*-aminoazotoluene would be expected to produce a high yield in the lungs.

FIG. 8.—The effects of *p*-dimethylaminoazobenzene on the kidney –SH levels of rats on a good quality diet.

Carbon tetrachloride has been found to cause an elevation of liver –SH levels in mice. Since this agent also causes fatty infiltration of the liver and all results have been calculated on the basis of wet weight of tissue the experimental results in this series will be effected by the presence of fat in the tissue. In fact, if allowance were made for this fat and the results were calculated as against weight of active liver tissue, the experimental figures would be higher than those recorded.

A very interesting set of figures in the present series is that obtained with *p*-dimethylaminoazobenzene in animals on a high quality diet. There was no distinction between the experimental and control groups, whereas earlier it was found that in animals on a poor (tumour promoting) diet there was a period when the livers of the experimental animals showed a higher –SH level than did the control livers. This suggests that with this agent any tissue –SH elevation is a consequence of dietary factors and is not due to the agent *per se*. Some evidence for this resides in the finding by Calcutt, Doxey and Coates (1961) that deletion of riboflavin from animals causes an elevation of liver –SH levels.

If the present results are taken together with those previously obtained by Calcutt *et al.* (1960) we have five different chemical carcinogens (3,4-benzopyrene, urethane, 2-fluoroenylacetamide, *o*-aminoazotoluene and carbon tetrachloride) which cause an elevation in the –SH levels of target tissues during the tumour induction period. None of these agents produced a similar –SH rise in non-target tissues. Similar, but not so well defined, results were obtained with *p*-dimethylaminoazobenzene, 1,2 : 5,6-dibenzacridine, and stilboestrol. To this can be added the finding by Boyland and Mawson (1938) that 1,2 : 5,6-dibenzocarbazole (a hepato-carcinogen) induces a large and prolonged rise in liver glutathione, whilst methylcholanthrene and 1,2 : 5,6-dibenzanthracene (neither affects the liver) had no such effect.

On the basis of the above data it is now possible to offer a tentative hypothesis in respect of the induction of tumours. It is already well recognised that tumour induction is a multi-stage process. It is now suggested that, apart from the agent causing initial cell damage, a necessary requirement for a tumour to appear is the elevation of the tissue –SH level above the normal level of the particular tissue. This elevation could arise directly as the result of application of the carcinogen as with 3,4-benzopyrene or *o*-aminoazotoluene or indirectly, as with *p*-dimethylaminoazobenzene where dietary restriction is necessary for tumour induction.

This hypothesis also offers a reasonable explanation for many findings in respect of anticarcinogens. Crabtree (1944, 1945, 1946) found that mercapturate forming agents and various other compounds known to inhibit –SH groups acted as anticarcinogens in respect of the induction of skin tumours by polycyclic hydrocarbons. This activity was believed to reside in the fact that combination of the hydrocarbon with tissue –SH groups was an essential for tumour formation and that prevention of this reduced the chance of a tumour occurring. There has, however, never been any substantiation of the idea of hydrocarbon/–SH binding. It can now be suggested that these anticarcinogens are active in that they inhibit an essential rise in tissue –SH levels.

So far the evidence in favour of the above views is little more than fragmentary, but at least the hypothesis offers a basis for further experimental work. Investigations of some further aspects of this problem are already in hand and will be reported shortly.

### SUMMARY

*o*-Aminoazotoluene has been found to induce a rise in –SH levels in mouse liver and lung but not kidney. Carbon tetrachloride causes an elevation of mouse liver –SH levels but not those of kidney.

1,2 : 5,6-Dibenzacridine causes a slight rise in mouse skin –SH levels.

*p*-Dimethylaminoazobenzene does not affect mouse liver –SH levels if the animals receive a high quality diet.

On the basis of this work and other evidence in the literature it is suggested that an elevation of tissue –SH levels is an essential prerequisite for tumour formation.

The expenses of this work were defrayed from a block grant by the British Empire Cancer Campaign.

### REFERENCES

ANDERVONT, H. B.—(1947) *J. nat. Cancer Inst.*, **7**, 431.
BOYLAND, E. AND MAWSON, ELINOR H.—(1938) *Biochem. J.*, **32**, 1460.
CALCUTT, G. AND DOXEY, D.—(1959) *Exp. Cell. Res.*, **17**, 542.
*Iidem* AND COATES, JOAN.—(1960) *Brit. J. Cancer*, **14**, 746.—(1961) *Nature, Lond.*, In press.
CRABTREE, H. G.—(1944) *Cancer Res.*, **4**, 688.—(1945) *Ibid.*, **5**, 346.—(1946) *Ibid.*, **6**, 553.
HARTWELL, J. L.—(1951) ' Survey of Compounds which have been Tested for Carcinogenic Activity '. 2nd Ed. Bethesda (National Cancer Institute).